# Cross‐sectional study on university students' mental health during the COVID‐19 pandemic: Exploring the influence of adverse and positive childhood experiences

**DOI:** 10.1002/pcn5.235

**Published:** 2024-08-15

**Authors:** Tetsuro Noda, Hiromu Nagaura, Yoshinobu Fujita, Toshihiko Tsutsumi

**Affiliations:** ^1^ Higashi Fuse Noda Clinic Higashiosaka Osaka Japan; ^2^ Osaka University of Human Science Settsu Osaka Japan; ^3^ Hokkaido University of Education Asahikawa‐City Hokkaido Japan; ^4^ University of Human Arts and Sciences Saitama‐City Saitama Japan

**Keywords:** adverse childhood experiences, depressive/anxiety symptoms, distress, positive childhood experiences, university students

## Abstract

**Aim:**

This study examined the psychological impact of the COVID‐19 pandemic on university students, focusing on how adverse childhood experiences (ACEs) and positive childhood experiences (PCEs) influence mental health.

**Methods:**

A web‐based survey was administered to 3000 university students from October 26 to 31, 2022, following the peak of the COVID‐19 pandemic. Mental health assessments included the Japanese version of the Kessler Psychological Distress 6‐Item Scale (K6) for depressive/anxiety symptoms, the Impact of Event Scale‐Revised (IES‐R‐J) for distress, fear of COVID‐19, and a three‐item loneliness scale.

**Results:**

Of the respondents, 46.9% reported depressive/anxiety symptoms, 55.4% reported distress, and 37.3% reported fear of COVID‐19. Factors such as current psychiatric treatment history and reduced income (either parental or personal) were predictive of worsening depressive/anxiety symptoms, distress, and loneliness. ACEs were found to exacerbate depressive/anxiety symptoms and distress, while PCEs mitigated these symptoms, and vice versa.

**Conclusion:**

This study underscores the importance of considering both ACEs and PCEs in supporting the mental health of university students. PCEs were found to independently prevent mental health deterioration, including depressive/anxiety symptoms and distress, which may include post‐traumatic stress disorder symptoms, even in the presence of ACEs. Recognizing and fostering PCEs emerged as an effective strategy for mitigating mental health issues.

## INTRODUCTION

Coronavirus disease 2019 (COVID‐19) is an emerging infectious disease caused by severe acute respiratory syndrome coronavirus (SARS‐CoV‐2). Initially detected in Wuhan, China, in December 2019, it has since led to numerous infections and fatalities. In Japan, preventive measures against the COVID‐19 pandemic have been enforced through the declaration of a state of emergency and prioritized actions to curb its spread. Mandates such as mask‐wearing, avoiding crowded spaces, and maintaining social distancing have been advocated to prevent transmission. This global health crisis has triggered various psychological responses, including traumatic stress symptoms, depression, anxiety, confusion, anger, insomnia, and suicidal ideation, with students being particularly susceptible to mental health challenges.^1^, ^2^

Since the onset of the COVID‐19 pandemic, Japan has witnessed a rise in suicides, particularly among women and young individuals.^3^ This trend suggests a potential exacerbation of mental health issues due to the pandemic. Students, in particular, have faced unique challenges, including isolation resulting from university closures and the prioritization of online classes. Noda et al.^4^ conducted a survey in May 2020, during the initial state of emergency (April 7 to May 25, 2020), and 1 month thereafter, revealing that at least 50% of respondents exhibited mild or greater depressive/anxiety symptoms according to the Japanese version of the Kessler Psychological Distress 6‐Item Scale (K6).^5^

There is ongoing debate regarding whether the COVID‐19 pandemic qualifies as a traumatic stressor leading to post‐traumatic stress disorder (PTSD), as per the *Diagnostic and Statistical Manual of Mental Illnesses* (DSM‐5)^6^ guidelines, which indicate that a life‐threatening or debilitating medical illness may not necessarily be considered a traumatic event.^7^ However, a substantial body of research supports the perspective that COVID‐19 can indeed be construed as a traumatic stressor, eliciting PTSD‐like reactions and exacerbating other related mental health issues.^8^, ^9^ We adopted this perspective to investigate PTSD symptoms among students. This study used the Japanese version of the Impact of Event Scale‐Revised (IES‐R‐J)^10^ to assess distress that may include PTSD symptoms.

In a study examining adverse childhood experiences (ACEs) in the United States during the 1990s, events such as familial abuse and mental illness experienced by individuals up to age 18 years were deemed traumatic, correlating with chronic diseases, mental health disorders, and substance abuse in adolescence and beyond.^11^ Among college students, higher ACE levels correlate with increased risk of mental health decline over time.^12^ ACEs have been shown to impact students' mental health during COVID‐19 lockdowns.^13^ Concurrently, during the pandemic, a greater number of ACEs may pose a risk for poorer mental health outcomes.

Conversely, higher levels of positive childhood experiences (PCEs), such as growing up with a supportive caregiver, having close friendships, and maintaining a predictable home routine, predict better mental health in adulthood.^14^, ^15^

Additionally, research has shown that, during the pandemic, aside from ACEs, the presence of PCEs, including having supportive caregivers or friends, is associated with decreased depressive symptoms, stress, and loneliness.^16^ However, research on PCEs in Japan remains limited. Given concerns about the deteriorating mental health of students amid the ongoing pandemic, we continuously monitored their mental well‐being and explored risk factors for a decline in their mental health. Three years post the COVID‐19 outbreak in Japan, we examined the impact of ACEs and PCEs on students' mental health, along with factors such as COVID‐19 awareness and sense of coherence (SOC), as investigated by Noda et al.^4^ in their study on students' mental health during the pandemic. We hypothesized that PCEs would mitigate the effects of ACEs on depressive/anxiety symptoms, distress, fear of COVID‐19, and loneliness.

## METHODS

### Participants and procedure

A web survey of students was conducted via MyVoice.com Inc., from October 26 to October 31, 2022, following the COVID‐19 wave that occurred between July 1, 2022, and September 30, 2022. A total of 3000 students participated in the survey, with 1480 (49.3%) identifying as male, an equal number identifying as female, 20 (0.7%) reporting as neither sex, and 20 (0.7%) omitting a response. Respondents were incentivized with points redeemable for Amazon gift certificates and other items valued at approximately $4.

During this COVID‐19 wave, approximately 1.47 million individuals were infected, marking the highest number compared to previous waves. Notably, individuals in their teens and twenties accounted for about 40% of the total infections, with the Omicron BA5 strain being predominant. The age range of respondents spanned from 18 to 25 years, with a mean age (standard deviation [SD]) of 20.6 (1.5) years. Among participants, 163 (5.4%) reported currently receiving psychiatric treatment.

### MEASURES

#### Depressive/anxiety symptoms, distress, loneliness, and SOC

The K6,^5^ designed to screen for depressive/anxiety symptoms over the preceding 30 days, assessed students' mental health status. Participants rated various statements on a five‐point scale (0 = *not at all* to 4 = *daily*). Total scores ranged from 0 to 24 points (0–4: no symptoms; 5–9: mild symptoms; 10–12: moderate symptoms; and 13–24: severe symptoms).

The IES‐R‐J was employed to evaluate distress.^10^ This self‐report measure, aligned with the DSM‐IV definition of PTSD,^17^ assessed the difficulty of 22 items experienced after high‐stress events (eight intrusion items, eight avoidance, and six hyperarousal). Respondents rated the difficulty of each aspect over the past week on a five‐point scale ranging from 0 (*not at all*) to 4 (*extremely*), yielding a total score range of 0–88 points. PTSD is suspected with a score of 25 points or more.

To evaluate loneliness, we employed the Japanese version of the three‐item loneliness scale (TIL).^18^ This scale comprised three items: (1) I feel that I lack companionship, (2) I feel left out, and (3) I feel isolated from others. Respondents rated the degree of each loneliness aspect on a four‐point Likert scale (1 = *never* to 4 = *often*), resulting in a scale score range of 3–12.

We considered SOC as a measure of resilience and utilized the University of Tokyo Health Sociology version of the three‐item Sense of Coherence Scale (SOC‐3‐UTHS)^19^ to assess SOC. Developed based on Antonovsky's concept of SOC,^20^ this instrument employs a seven‐point scale to explore three dimensions: comprehensibility, manageability, and meaningfulness.

#### Demographic information and changes in COVID‐19 awareness

Participants provided demographic details including sex, age, residential area, university affiliation, grade, and current psychiatric treatment (see Table 1).

**Table 1:** Demographic characteristics (*N* = 3000).

Characteristic		*N*	%
Gender	Male	1480	49.3
	Female	1480	49.3
	Neither	20	0.7
	No answer	20	0.7
University establishing body	National	663	22.1
	Public	234	7.8
	Private	2103	70.1
Grade	1st year	623	20.8
	2nd year	702	23.4
	3rd year	665	22.2
	4th year＋	1010	33.7
Geographic area	Hokkaido	111	3.7
	Tohoku	171	5.7
	Kantou	1182	39.4
	Hokuriku	119	4.0
	Choubu	347	11.6
	Kinki	628	20.9
	Chougoku	160	5.3
	Shikoku	59	2.0
	Kyushu	223	7.4
Current psychiatric treatment	Yes	163	5.4
Age (SD), years		20.6	1.5

Abbreviation: SD, standard deviation.

Additionally, participants were queried about their COVID‐19 awareness and economic concerns, responding to statements such as “I feel afraid of COVID‐19,” “I feel stressed about changes in my daily life due to COVID‐19 prevention measures,” “I am worried about my future because of COVID‐19,” and “I feel insecure due to reduced income for myself or my guardian.” Using a five‐point Likert scale (1 = *strongly disagree*, 2 = *disagree*, 3 = *undecided*, 4 = *agree*, 5 = *strongly agree*) participants rated their sentiments over the past 30 days (see Table 2).

**Table 2:** Awareness of COVID‐19 and economic problems in the past 30 days (*N* = 3000).

	Strongly disagree	Disagree	Undecided	Agree	Strongly agree
Stressed by COVID‐19 prevention	968 (32.3)	629 (21.0)	526 (17.5)	717 (23.9)	160 (5.3)
Feeling uncertain about future due to COVID‐19	1040 (34.7)	577 (19.2)	594 (19.8)	604 (20.1)	185 (6.2)
I feel afraid of COVID‐19	716 (23.9)	538 (17.9)	626 (20.9)	841 (28.0)	279 (9.3)
Living in insecurity due to reduced income of my guardian or myself	1147 (38.2)	611 (20.4)	634 (21.1)	427 (14.2)	181 (6.0)

*Note*: *N* (%).

#### ACEs and PCEs

To assess ACEs, participants responded with *yes* or *no* to 10 items, inquiring whether they had experienced the following before the age of 18 years: (1) psychological abuse, (2) physical abuse, (3) sexual abuse, (4) psychological neglect, (5) parental divorce or separation, (6) family mental illness or suicide, (7) witnessing domestic violence, (8) family substance abuse, (9) abandonment, and (10) family member imprisonment.^21^, ^22^ The ACE score represented the count of items receiving a positive response.

For PCEs assessment, we employed the benevolent childhood experiences Scale, a 10‐item questionnaire developed by Narayan et al.^14^ and translated into Japanese with Professor Narayan's permission. To enhance comprehension among Japanese participants, we replaced “beliefs” with “places” in the item “Did you have beliefs that gave comfort.” Additionally, the original text was interpreted and translated into Japanese for better understanding. Participants responded with *yes* or *no* regarding experiences before the age of 18 years, including having a safe caregiver, a good friend, comforting places, enjoying school, a caring teacher, good neighbors, supportive adults, enjoyable activities, positive self‐concept, and predictable home routine. The PCEs score represented the count of items receiving a positive response.

Due to the sensitive nature of ACE and PCE questions, participants unwilling to answer were not obliged to do so. Results showed that 2713 (90.4%) respondents completed all ACE items (*M* = 0.56, SD = 1.37, range = 0–10). Among these respondents, 75.7% reported none of the items as applicable, while 0.5% reported all 10 items as applicable.

Regarding the PCEs Scale, 2575 (85.8%) respondents answered all items (*M* = 7.29, SD = 3.13, range = 0–10). Among these respondents, 7.8% reported none of the items as applicable, while 34.0% reported all 10 items as applicable.

#### Data analysis

Data were analyzed using SPSS Version 29.0. Pearson correlations were employed to examine the associations among the K6, IES‐R‐J, TIL, SOC‐3‐UTHS, ACEs, and PCEs. Subsequently, we investigated the significant relationships of ACEs, PCEs, and covariates with: (1) depressive/anxiety symptoms (K6), (2) distress (IES‐R‐J), (3) fear of COVID‐19, and (4) loneliness (TIL), using hierarchical logistic regression analysis. Dummy variables were established for the dependent variables. The cutoff points for K6 and IES‐R‐J were set at 9/10 and 24/25, respectively, with dummy variables assigned as 0 or values below the cutoff point and 1 for values above it. While TIL has been tested for reliability and validity, no cutoff point has been established. Therefore, loneliness was defined as a TIL score <7 = 0 and TIL score ≥7 = 1, given that the mean TIL score was 7 (rounded). Fear of COVID‐19 was designated as 0 if the respondent answered “I feel afraid of COVID‐19” with *strongly disagree*, *disagree*, or *undecided*, and 1 if the respondent answered *agree* or *strongly agree*. Covariates included sex, age, current psychiatric treatment, feelings of insecurity due to reduced income, and SOC‐3‐UTHS score.

To examine the effects of ACEs and PCEs on the four dependent variables, hierarchical logistic regression analysis was conducted with five covariates in the first step. ACE scores were added to the five covariates in the second step, and PCE scores were added in the third step. PCE scores were added to the five covariates in the fourth step. Missing values were only found for ACEs and PCEs, ranging from 2.2% to 3.3% of the data for each of the ACEs and from 2.6% to 6.1% of the data for each of the PCEs. In calculating Pearson correlations and performing hierarchical logistic analysis, we included 2462 participants who responded to both ACEs and PCEs.

## RESULTS

### Descriptive statistics

The Cronbach's *α* for K6 was 0.918. The K6 scores ranged from 0 to 24 points, with a mean (SD) of 5.56 (5.81). Among the participants, the level of depressive/anxiety symptoms was 23.1% mild, 10.3% moderate, and 13.4% severe. The IES‐R‐J scores ranged from 0 to 88 points, with a mean (SD) of 28.6 (20.0). The Cronbach's *α* for IES‐R‐J was 0.854. In addition, 55.4% of the participants reported severe distress (IES‐R‐J ≥ 25). TIL scores ranged from 3 to 12 points, with a mean (SD) of 7.14 (2.53). The Cronbach's *α* for TIL was 0.880. SOC‐3‐UTHS scores ranged from 3 to 21 points, with a mean (SD) of 13.28 (3.78). The Cronbach's *α* for SOC‐3‐UTHS was 0.846.

The ACE and PCE scores and item counts are displayed in Tables 3, 4, 5. The Cronbach's *α* for ACEs and PCEs were 0.801 and 0.897, respectively.

**Table 3:** Number of ACEs and PCEs applicable.

	ACEs		PCEs	
	*N*	%	*N*	%
0	2053	68.4	201	6.7
1	328	10.9	47	1.6
2	127	4.2	45	1.5
3	83	2.8	80	2.7
4	40	1.3	99	3.3
5	35	1.2	143	4.8
6	18	0.6	171	5.7
7	11	0.4	218	7.3
8	3	0.1	273	9.1
9	2	0.1	423	14.1
10	13	0.4	875	29.2
NA	287	9.6	425	14.2

Abbreviations: ACEs, adverse childhood experiences; NA, no answer; PCEs, positive childhood experiences.

**Table 4:** Frequencies of ACEs.

ACEs	Yes (%)	No (%)	NA (%)
1. Physical abuse	6.1	90.8	3.1
2. Psychological abuse	8.9	87.8	3.2
3. Sexual abuse	4.4	92.3	3.3
4. Psychological neglect	9.1	87.9	3.0
5. Family alcohol/drug dependence	4.1	93.9	2.0
6. Domestic violence in front of children	8.5	88.6	2.9
7. Family mental illness and suicide	6.7	91.1	2.2
8. Divorce or separation of parents	10.8	86.8	2.4
9. Abandoned childcare	3.8	93.9	2.3
10. Family imprisonment	2.1	95.7	2.2

Abbreviations: ACEs, adverse childhood experiences; NA, no answer.

**Table 5:** Frequencies of PCEs.

PCEs	Yes (%)	No (%)	NA (%)
1. Having at least one safe caregiver	75.9	20.3	3.8
2. Having at least one good friend	82.3	15.0	2.7
3. Having places that gave comfort	77.9	18.9	3.3
4. Enjoying school	58.8	36.9	4.3
5. Having at least one teacher who cared	70.2	26.0	3.8
6. Having good neighbors	57.5	38.8	3.7
7. Having an adult (not a parent/caregiver) who could provide support or advice	64.6	32.0	3.4
8. Having opportunities to have a good time	82.4	14.4	3.2
9. Having a positive self‐concept	52.8	41.1	6.1
10. Having a predictable home routine	63.4	34.1	2.6

Abbreviations: NA, no answer; PCEs, positive childhood experiences.

Bivariate correlations between the variables are presented in Table 6. High correlations were observed between K6 and IES‐R‐J (*r* = 0.520, *p* < 0.01), and K6 and TIL (*r* = 0.506, *p* < 0.01). ACEs and PCEs exhibited a slight negative correlation (*r* = −0.119, *p* < 0.01). PCEs displayed a modest positive correlation with SOC‐3‐UTHS (*r* = 0.313, *p* < 0.01).

**Table 6:** Bivariate correlations between variables.

	1	2	3	4	5	6
1. K6	ー					
2. IES‐R‐J	0.520**	ー				
3. TIL	0.506**	0.338**	ー			
4. SOC‐3‐UTHS	−0.060**	−0.004	−0.042*	ー		
5. ACEs	0.219**	0.181**	0.106**	−0.051**	ー	
6. PCEs	−0.218**	−0.201**	−0.119**	0.313**	−0.119**	ー

Abbreviations: ACEs, adverse childhood experiences; IES‐R‐J, Japanese version of the Impact of Event Scale‐Revised; K6, Kessler Psychological Distress 6‐Item Scale; PCEs, positive childhood experiences; SOC‐3‐UTHS, University of Tokyo Health Sociology version of the three‐item Sense of Coherence Scale; TIL, Japanese version of the three‐item loneliness scale.

*p < 0.05. **p < 0.01.

### Hierarchical logistic regression analysis

Table 7 displays the results of the hierarchical binomial logistic analysis. The omnibus tests for each model were significant (*p* < 0.001).

**Table 7:** Hierarchical binomial logistic regression for depressive/anxiety symptoms, distress, fear of COVID‐19, and loneliness.

	Depressive/anxiety symptoms (K6)			Distress (IES‐R‐J)			Fear of COVID‐19			Loneliness(TIL)		
	*p* value	OR	95% CI	*p* value	OR	95% CI	*p* value	OR	95% CI	*p* value	OR	95% CI
Step 1. Covariates												
Gender												
Male	Reference			Reference			Reference			Reference		
Female	0.009	1.32	[1.07–1.63]	0.962	1.00	[0.85–1.17]	<0.001	1.53	[1.29–1.82]	0.020	1.22	[1.03–1.43]
Neither	0.01	4.21	[1.40–12.62]	0.106	2.64	[0.81–8.55]	0.240	1.92	[0.65–5.68]	0.867	0.91	[0.32–2.63]
No answer	0.504	1.59	[0.41–6.13]	0.181	2.28	[0.68–7.65]	0.182	2.12	[0.70–6.38]	0.461	1.53	[0.49–4.75]
Age	0.143	0.95	[0.88–1.02]	0.013	0.93	[0.88–0.99]	0.381	0.97	[0.92–1.03]	0.046	0.94	[0.89–1.00]
Current psychiatric treatment												
No	Reference			Reference			Reference			Reference		
Yes	<0.001	4.57	[3.06–6.84]	<0.001	3.23	[2.04–5.12]	0.018	1.62	[1.09–2.41]	<0.001	3.01	[1.86–4.86]
Living in insecurity due to reduced income of my guardian or myself												
Disagree	Reference			Reference			Reference			Reference		
Undecided	<0.001	2.99	[2.33–3.84]	<0.001	2.34	[1.90–2.90]	<0.001	1.56	[1.25–1.94]	<0.001	1.56	[1.27–1.93]
Agree	<0.001	3.86	[3.00–4.97]	<0.001	2.56	[2.05–3.19]	<0.001	3.72	[2.98–4.65]	<0.001	2.80	[2.22–3.54]
SOC‐3‐UTHS	<0.001	0.95	[0.92–0.98]	0.141	0.98	[0.96–1.01]	<0.01	1.09	[1.07–1.12]	0.773	1.00	[0.98–1.02]
	Cox–Snell *R* ^2^/Nagelkerke *R* ^2^ = 0.09/0.15			Cox–Snell *R* ^2^/Nagelkerke *R* ^2^ = 0.06/0.08			Cox–Snell *R* ^2^/Nagelkerke *R* ^2^ = 0.09/0.13			Cox–Snell *R* ^2^/Nagelkerke *R* ^2^ = 0.05/0.07		
Step 2 ACEs												
Gender												
Male	Reference			Reference			Reference			Reference		
Female	0.006	1.34	[1.09–1.66]	0.971	1.00	[0.85–1.18]	<0.001	1.53	[1.29–1.83]	0.019	1.22	[1.03–1.44]
Neither	0.011	4.22	[1.39–12.80]	0.113	2.61	[0.80–8.52]	0.232	1.94	[0.66–5.73]	0.857	0.91	[0.32–2.62]
No answer	0.468	1.65	[0.43–6.33]	0.181	2.28	[0.68–7.64]	0.183	2.12	[0.70–6.38]	0.459	1.53	[0.49–4.76]
Age	0.139	0.95	[0.88–1.02]	0.011	0.93	[0.88–0.98]	0.396	0.98	[0.92–1.03]	0.044	0.94	[0.89–1.00]
Current psychiatric treatment												
No	Reference			Reference			Reference			Reference		
Yes	<0.001	3.94	[2.60–5.97]	<0.001	2.88	[1.81–4.59]	0.009	1.71	[1.14–2.56]	<0.001	2.90	[1.77–4.65]
Living in insecurity due to reduced income of my guardian or myself												
Disagree	Reference			Reference			Reference			Reference		
Undecided	<0.001	3.08	[2.39–3.97]	<0.001	2.38	[1.92–2.95]	<0.001	1.56	[1.25–1.94]	<0.001	1.57	[1.27–1.93]
Agree	<0.001	3.62	[2.81–4.67]	<0.001	2.43	[1.94–3.04]	<0.001	3.81	[3.04–4.77]	<0.001	2.75	[2.17–3.48]
SOC‐3‐UTHS	0.001	0.96	[0.93–0.98]	0.221	0.99	[0.97–1.01]	<.001	1.09	[1.07–1.12]	0.855	1.00	[0.98–1.02]
ACEs	<0.001	1.25	[1.16–1.33]	<0.001	1.20	[1.12–1.29]	0.106	0.95	[0.88–1.01]	0.060	1.07	[1.00–1.14]
	Cox–Snell *R* ^2^/Nagelkerke *R* ^2^ = 0.11/0.17			Cox–Snell *R* ^2^/Nagelkerke *R* ^2^ = 0.07/0.10			Cox–Snell *R* ^2^/Nagelkerke *R* ^2^ = 0.09/0.13			Cox–Snell *R* ^2^/Nagelkerke *R* ^2^ = 0.05/0.07		
Step 3 ACE ＋ PCEs												
Gender												
Male	Reference			Reference			Reference			Reference		
Female	0.005	1.36	[1.10–1.69]	0.924	0.99	[0.84–1.17]	<0.001	1.54	[1.30–1.84]	0.020	1.22	[1.03–1.44]
Neither	0.021	3.71	[1.22–11.28]	0.185	2.24	[0.68–7.35]	0.185	2.11	[0.70–6.34]	0.809	0.88	[0.31–2.53]
No answer	0,292	2.06	[0.54–7.87]	0.128	2.55	[0.76–8.54]	0.223	1.99	[0.66–6.02]	0.433	1.57	[0.51–4.87]
Age	0.054	0.93	[0.87–1.00]	0.002	0.92	[0.86–0.97]	0.521	0.98	[0.92–1.04]	0.034	0.94	[0.89–1.00]
Current psychiatric treatment												
No	Reference			Reference			Reference			Reference		
Yes	<0.001	3.91	[2.57–5.95]	<0.001	2.81	[1.76–4.50]	0.007	1.75	[1.17–2.62]	<0.001	2.85	[1.75–4.62]
Living in insecurity due to reduced income of my guardian or myself												
Disagree	Reference			Reference			Reference			Reference		
Undecided	<0.001	2.81	[2.17–3.64]	<0.001	2.23	[1.80–2.78]	<0.001	1.64	[1.31–2.04]	<0.001	1.54	[1.25–1.90]
Agree	<0.001	3.43	[2.65–4.44]	<0.001	2.31	[1.85–2.90]	<0.001	3.99	[3.18–5.01]	<0.001	2.71	[2.14–3.43]
SOC‐3‐UTHS	0.485	0.99	[0.96–1.02]	0.178	1.02	[0.99–1.04]	<0.001	1.08	[1.05–1.11]	0.720	1.00	[0.98–1.03]
ACEs	<0.001	1.22	[1.14–1.31]	<0.001	1.17	[1.09–1.25]	0.261	0.96	[0.90–1.03]	0.085	1.06	[0.99–1.13]
PCE	<0.001	0.87	[0.84–0.90]	<0.001	0.89	[0.87–0.92]	<0.001	1.07	[1.03–1.10]	0.092	0.98	[0.95–1.00]
	Cox–Snell *R* ^2^/Nagelkerke *R* ^2^ = 0.13/0.20			Cox–Snell *R* ^2^/Nagelkerke *R* ^2^ = 0.10/0.13			Cox–Snell *R* ^2^/Nagelkerke *R* ^2^ = 0.10/0.14			Cox–Snell *R* ^2^/Nagelkerke *R* ^2^ = 0.05/0.07		
Step 4 PCEs												
Gender												
Male	Reference			Reference			Reference			Reference		
Female	0.006	1.34	[1.09–1.66]	0.897	0.99	[0.84–1.17]	<0.001	1.54	[1.30–1.84]	0.020	1.22	[1.03–1.43]
Neither	0.021	3.68	[1.22–11.16]	0.181	2.25	[0.69–7.36]	0.188	2.1	[0.70–6.33]	0.813	0.88	[0.31–2.53]
No answer	0.304	2.02	[0.53–7.76]	0.127	2.56	[0.77–8.53]	0.223	1.99	[0.70–6.00]	0.432	1.57	[0.51–4.88]
Age	0.054	0.932	[0.87–1.00]	0.003	0.92	[0.87–0.97]	0.515	0.98	[0.92–1.04]	0.035	0.941	[0.89–1.00]
Current psychiatric treatment												
No	Reference			Reference			Reference			Reference		
Yes	<0.001	4.45	[2.98–6.74]	<0.001	3.09	[1.94–4.92]	0.011	1.69	[1.13–2.52]	<0.001	2.97	[1.83–4.80]
Living in insecurity due to reduced income of my guardian or myself												
Disagree	Reference			Reference			Reference			Reference		
Undecided	<0.001	2.74	[2.22–3.53]	<0.001	2.19	[1.77–2.73]	<0.001	1.64	[1.32–2.05]	<0.001	1.53	[1.24–1.90]
Agree	<0.001	3.64	[2.82–4.69]	<0.001	2.40	[1.92–3.01]	<0.001	3.93	[3.14–4.93]	<0.001	2.76	[2.18–3.45]
SOC‐3‐UTHS	0.39	0.99	[0.96–1.02]	0.212	1.02	[0.99–1.04]	<0.001	1.08	[1.05–1.11]	0.751	1	[0.98–1.03]
PCEs	<0.001	0.87	[0.84–0.90]	<0.001	0.89	[0.86–0.91]	<0.001	1.07	[1.04–1.10]	0.063	0.97	[0.95–1.00]
	Cox–Snell *R* ^2^/Nagelkerke *R* ^2^ = 0.12/0.19			Cox–Snell *R* ^2^/Nagelkerke *R* ^2^ = 0.09/0.12			Cox–Snell *R* ^2^/Nagelkerke *R* ^2^ = 0.10/0.13			Cox–Snell *R* ^2^/Nagelkerke *R* ^2^ = 0.05/0.07		

Abbreviations: ACEs, adverse childhood experiences; IES‐R‐J, Japanese version of the Impact of Event Scale‐Revised; K6, Kessler Psychological Distress 6‐Item Scale; PCEs, positive childhood experiences; SOC‐3‐UTHS, University of Tokyo Health Sociology version of the three‐item Sense of Coherence Scale; TIL, Japanese version of the three‐item loneliness scale.

#### Depressive/anxiety symptoms

In Step 1, sex, current psychiatric treatment, “Living in insecurity due to reduced income of my guardian or myself,” and SOC‐3‐UTHS were significantly associated with depressive/anxiety symptoms. In Step 2, along with these covariates, the ACEs scores also showed significance with an odds ratio (OR) of 1.25 (95% confidence interval [CI] [1.16–1.33]). The addition of PCEs, alongside the previous independent variables in Step 3, revealed significant differences (OR = 0.87, 95% CI [0.84–0.90] for PCEs). Adding PCEs with covariates in Step 4 also revealed a significant difference (OR = 0.87, 95% CI [0.84–0.90]). Using Step 3 as a model, the predictors of worse depressive/anxiety symptoms were as follows: female or neither versus male (OR = 1.31, 95% CI [1.10–1.69], OR = 3.71, 95% CI [1.22–11.28], respectively). Agree and *undecided* responses to “Living in insecurity due to reduced income of my guardian or myself” and receiving psychiatric treatment had stronger impacts on depressive/anxiety symptoms. Higher ACEs scores were associated with greater depressive/anxiety symptoms, whereas higher PCEs scores were associated with the opposite trend.

#### Distress

In Step 1, age, current psychiatric treatment, and “Living in insecurity due to reduced income of my guardian or myself” were significantly associated with severe distress. In Step 2, along with these covariates, the ACEs scores also demonstrated significance with an OR of 1.20 (95% CI [1.12–1.29]). When PCEs were added in Step 3, they showed a significant relationship (OR = 0.89, 95% CI [0.87–0.92]). The addition of PCEs with covariates in Step 4 also revealed a significant difference (OR = 0.89, 95% CI [0.86–0.91]). Using Step 3 as a model, the predictors of worsening distress were younger age, receiving psychiatric treatment, and an agree or *undecided* response to “Living in insecurity due to reduced income of my guardian or myself.” Higher ACEs scores were associated with greater distress, whereas higher PCEs scores were associated with decreased distress.

#### Fear of COVID‐19

In Step 1, sex, current psychiatric treatment, “Living in insecurity due to reduced income of my guardian or myself,” and SOC‐3‐UTHS were significantly related to fear of COVID‐19. When ACEs were added in Step 2, they did not demonstrate a significant relationship with fear of COVID‐19 (OR = 0.95, 95% CI [0.88–1.01]). In Step 3, when PCEs were added, PCEs showed a significant relationship with fear of COVID‐19 (OR = 1.07, 95% CI [1.03–1.10]). The addition of PCEs with covariates in Step 4 also revealed a significant difference (OR = 1.07, 95% CI [1.04–1.10]).

Using Step 3 as the model, the predictors of exacerbating fear of COVID‐19 were female sex, receiving psychiatric treatment, an agree or *undecided* response to “Living in insecurity due to reduced income of my guardian or myself,” higher SOC‐3‐UTHS score, and higher PCEs score.

#### Loneliness

In Step 1, sex, age, current psychiatric treatment, and “Living in insecurity due to reduced income of my guardian or myself” were significantly associated with loneliness. In Step 2, when ACEs were added, the covariates that were significantly related to loneliness in Step 1 remained significant; however, the relationship between ACEs and loneliness was not significant (OR = 1.07, 95% CI [1.00–1.09]).

When PCEs were added in Step 3, the covariates with a significant relationship with loneliness in Step 2 remained significant. PCEs did not show a significant relationship with loneliness (OR = 0.98, 95% CI [0.95–1.00]). The addition of PCEs with covariates in Step 4 revealed no significant difference (OR = 0.97, 95% CI [0.95–1.00]).

Using Step 3 as the model, females were more likely to experience loneliness than males (OR = 1.22, 95% CI [1.03–1.44]). Greater loneliness was also associated with receiving current psychiatric treatment and an agree or *undecided* response to “Living in insecurity due to reduced income of my guardian or myself.”

## DISCUSSION

In this study, 46.9% of the participants reported K6 scores ≥5, indicating depressive/anxiety symptoms, while 13.4% reported scores ≥13, suggesting severe depressive/anxiety symptoms. An online survey assessing 11,333 members of the general Japanese population (mean age [SD] = 46.3 [14.6]) after the first state of emergency found that 48.1% had K6 scores ≥5, with 11.5% having scores ≥13, indicating a higher prevalence of severe symptoms among younger individuals.^23^ In the 2019 Comprehensive Survey of Living Conditions,^24^ approximately 30% of respondents over the age of 20 years had a K6 score ≥5, suggesting that the mental health impact of the COVID‐19 pandemic extends beyond students to the entire nation.

Ochnik et al.^2^ conducted a study using a PTSD checklist specific version among students across several European countries during the second wave of the COVID‐19 pandemic (mid‐October to December 2020) and found that 78.2% of respondents exhibited suspected PTSD with the pandemic as a stressor. In studies not limited to specific stressors, 42.9% of students in France were symptom‐positive according to the IES‐R conducted shortly after the peak mortality rate in France in April 2020.^25^ Similarly, in a study conducted in Spain over an 8‐day period starting on March 28, 2020, 50.4% of students were symptom‐positive according to the original IES.^26^ In our study, using the IES‐R‐J in students without limiting stressors, 55.4% were found to be at risk of severe distress, underscoring the significant impact of the COVID‐19 pandemic on students' mental health.

Both PCEs and ACEs play crucial roles in shaping individuals' development throughout their lives.^14^, ^15^, ^16^, ^27^, ^28^, ^29^ In our study, we chose to focus on PCEs and their relationship with students' mental health outcomes. Our hierarchical logistic analysis revealed that higher scores on ACEs were associated with increased depressive/anxiety symptoms and distress. Conversely, higher scores on PCEs were associated with decreased depressive/anxiety symptoms and distress, although they were linked to an increased fear of COVID‐19. Experiencing fear during a pandemic can be viewed as a natural response aimed at taking appropriate measures to safeguard against infection. Therefore, PCEs may contribute to fostering a sense of resilience and adaptive crisis awareness during emergencies like the COVID‐19 pandemic.

Regarding our hypotheses, we confirmed that PCEs indeed mitigated the effects of ACEs on depressive/anxiety symptoms and distress. However, we found that ACEs did not influence the fear of COVID‐19, while PCEs had an exacerbating effect on this fear. Additionally, neither ACEs nor PCEs showed any effect on loneliness in our study. Interestingly, while our study did not observe a direct effect of ACEs and PCEs on loneliness, previous research has demonstrated that ACEs heightened students' loneliness while PCEs reduced it.^30^

The relationships among ACEs, PCEs, and loneliness remain relatively unexplored in the literature, indicating a potential avenue for future research. In a systematic review of 58 studies on the impact of PCEs on adult outcomes, Han et al.^29^ concluded that PCEs independently predict more favorable outcomes without interacting with or moderating ACEs. Moreover, PCEs are deemed crucial targets for fostering resilience in adults, particularly among those who have experienced significant childhood adversity. Noda et al.^4^ reported that a higher SOC, indicative of resilience, correlates with reduced depressive/anxiety symptoms. However, our current study did not find a significant effect of SOC on depressive/anxiety symptoms when PCEs were included in the model. Interestingly, while Step 1 and Step 2 of our analysis did not reveal a significant relationship between SOC and distress, the inclusion of PCEs led to a reduction in distress. Previous studies^31^ have indicated that a strong SOC reduces PTSD symptoms, but our findings are not consistent with these findings. The correlation coefficients between depressive/anxiety symptoms or distress and SOC were low, indicating that as the COVID‐19 pandemic progresses, the influence of SOC may diminish, while the impact of ACEs and PCEs, which have a more substantial effect, may become more prominent.

A trend was observed indicating that neither males nor females exhibited depressive/anxiety symptoms of moderate or greater levels, highlighting the importance of focusing on the mental health of individuals with gender dysphoria.

## LIMITATIONS

There are several limitations to consider in this survey. First, the online nature of the survey may have introduced bias towards participants with computer skills. Second, the use of screening tools rather than face‐to‐face diagnosis may have led to an overestimation of the frequency of depressive/anxiety symptoms and distress. Additionally, since the questionnaires were self‐administered, variations in respondents' language abilities and comprehension of the questionnaire could have influenced the results. Furthermore, there may have been a tendency towards central responses due to limited alternative options in the survey questions. Despite these limitations, the findings of this study contribute valuable insights into the mental health of students during the COVID‐19 pandemic.

## CONCLUSION

Roughly 47% of students displayed depressive/anxiety symptoms, while approximately 55% exhibited severe distress, which may include PTSD symptoms. Current psychiatric treatment history, experiencing reduced income (either parental or personal), and suspicion of gaming disorder were identified as predictors of exacerbated depressive/anxiety symptoms, distress, and loneliness. Paying attention to these factors is crucial to prevent the deterioration of students' mental health during the COVID‐19 pandemic.

Further, PCEs emerged as predictors of reduced depressive/anxiety symptoms and distress. While ACEs are typically associated with mental health risks, such as depressive/anxiety symptoms and distress, this study revealed that PCEs can independently prevent mental health decline, even in the presence of ACEs. PCEs encompass positive childhood experiences at home, in the community, and at school, emphasizing the importance of nurturing environments in fostering good mental health, even amid global crises like the COVID‐19 pandemic. Recognizing and promoting PCEs were found to be effective strategies in mitigating mental health challenges.

## AUTHOR CONTRIBUTION

**Tetsuro Noda:** Conceptualization; data curation; formal analysis; project administration; writing—original draft; writing—review and editing. **Hiromu Nagaura:** Conceptualization; data curation; formal analysis; writing—review and editing. **Yoshinobu Fujita:** Conceptualization; data curation; formal analysis. **Toshihiko Tsutsumi:** Writing—reviewing and editing. All authors read and approved the final draft.

## CONFLICT OF INTEREST STATEMENT

The authors declare no conflict of interest.

## ETHICS APPROVAL STATEMENT

The ethics committee of Osaka University of Human Science approved this study (No. 2022‐5).

## PATIENT CONSENT STATEMENT

All the participants were informed of the purpose of the study. It was explained that participation was anonymous and voluntary and informed written consent was provided by all participants.

## CLINICAL TRIAL REGISTRATION

N/A

## Data Availability

Data are available upon reasonable request.
